# A Comparison Between Two-Dimensional and Three-Dimensional Regional and Global Longitudinal Strain Echocardiography to Evaluate Complex Coronary Lesions in Patients With Non-ST-Segment Elevation Acute Coronary Syndrome

**DOI:** 10.7759/cureus.24025

**Published:** 2022-04-11

**Authors:** Mahmoud Raslan, Khaled A Elkhashab, Mohamed G Mousa, Yazid A Alghamdi, Haytham S Ghareb

**Affiliations:** 1 Cardiovascular Medicine, King Fahd Hospital of the University, Al Khobar, SAU; 2 Cardiology, Fayoum University Hospital, Fayoum, EGY; 3 Cardiology, Fayoum University, Fayoum, EGY; 4 Medicine, Imam Abdulrahman Bin Faisal University, Al Khobar, SAU

**Keywords:** syntax score, acute coronary syndrome, area strain, radial strain, circumferential strain, global longitudinal strain, speckle tracking echocardiography

## Abstract

Introduction

A preliminary assessment of patients who suffer from severe and complex coronary artery lesions, such as three-vessel disease and/or a left main (LM) artery lesion, plays a critical contribution in determining prognosis and treatment plans for non-ST-segment elevation acute coronary syndrome (NSTE-ACS). Therefore, a pre-angiography (i.e., before angiography) predictor was required to cost-effectively evaluate severe and complex coronary lesions to efficiently direct our subsequent dealing.

Aim

This study aimed to compare two-dimensional (2D) and three-dimensional (3D) global longitudinal strain (GLS) at the regional level to assess extremely complicated coronary lesions using the SYNTAX score as a standard of reference in 100 patients with NSTE-ACS.

Materials and methods

This research included 100 patients with non-ST-segment elevation acute coronary syndrome who presented at the Cardiology Department at Fayoum University from December 2019 to July 2020. All patients underwent a complete history and physical examination, hemoglobin A1c (HbA1c), lipid profile, creatinine assessment, 12-lead electrocardiogram (ECG), and transthoracic echocardiography (TTE) to detect global and regional longitudinal strain by 2D and 3D speckle-tracking echocardiography (STE). Coronary angiography was done on all patients within 24 hours of admission after acquiring echo images. Then, the results of 2D and 3D regional and global longitudinal strain (GLS) to predict the severity and coronary lesion complexity in terms of the SYNTAX score were compared.

Results

This study revealed that 2D GLS was −12.10 ± 3.51, which is significantly higher than 3D GLS of −11.64 ± 4.05 (p < 0. 001). The left anterior descending coronary artery (LAD) and left circumflex artery (LCX) territories revealed a significantly higher value using 2D regional longitudinal strain (−11.13 ± 4.47 and −12.54 ± 4.11, respectively) than using 3D regional longitudinal strain (−10.84 ± 5.18 and −12.05 ± 4.29, respectively) (p= 0.017 and p < 0.001, respectively). There were significantly lower 2D GLS, 3D GLS, global circumferential strain (GCS), area strain, and global radial strain (GRS) in the intermediate and high score group than in the low score group of patients (p < 0.001 for all).

Conclusion

2D and 3D strain echocardiography including GLS, GCS, GRS, and area strain are a noninvasive and rapid tool with clinical utility for evaluating coronary lesions in patients with NSTE-ACS. They can be routinely used to diagnose and stratify high-risk patients with NSTE-ACS, thereby potentially resulting in improved patient assessment. GLS as measured by 2D and 3D STE at minimal effort is a significant risk factor for patients with complex NSTE-ACS. In NSTE-ACS cases, the GLS absolute value is significantly associated with the degree of complexity of coronary artery lesions.

## Introduction

The acute estimation of cases with severe and complex coronary artery disease (CAD), such as three-vessel disease and/or a left main (LM) lesion, presents critical participation in determining prognosis and treatment plan for patients with non-ST-segment elevation acute coronary syndrome (NSTE-ACS) [1].

Clinical guidelines for NSTE-ACS management recently highly suggested initiating dual antiplatelet therapy as soon as possible with aspirin and one of the P2Y12 inhibitors including clopidogrel, ticagrelor, or prasugrel [2]. However, dual antiplatelet therapy may raise the risk of perioperative bleeding in patients undergoing early coronary artery bypass graft (CABG) surgery.

Therefore, clinicians may withdraw P2Y12 inhibitors in cases of high risk of requiring CABG. Soon, the administration of double antiplatelet therapy would necessitate postponement of CABG due to concerns of intraoperative bleeding. However, delaying the treatment with these medications may lead to an increased risk of fatal situations in patients with NSTE-ACS [3].

Thus, we require a single early (i.e., before angiography) indicator to cost-effectively evaluate coronary artery lesions that are severe and complex to efficiently lead our subsequent treatment. The SYNTAX trial elucidated the synergy between Taxus-based percutaneous coronary intervention and cardiac surgery (SYNTAX score) [1]. It was described as “an instrument for measuring the coronary artery’s disease complexity via angiography” [4].

SYNTAX is an extensive angiographic scoring system due to the complex nature of lesions in the coronary arteries [5]. The SYNTAX score can be used to determine the severity of coronary artery stenosis and evaluate the presence of bifurcation-, calcification-, tortuosity-, or trifurcation-type coronary artery lesions. However, it is an invasive procedure based on coronary angiography.

Significant coronary artery stenosis may result in chronically impaired longitudinal LV function at rest; thus, two-dimensional (2D) speckle-tracking echocardiography (STE) is better than the conventional 2D echocardiography in assessing myocardial function on a regional and global scale, as well as in determining the size of infarcts, the viability of the myocardium that has been infarcted, and mild variations associated with myocardial ischemia [6]. STE is a semiautomated mode of operation; thus, it has a high level of intra- and interobserver reproducibility [7].

STE is a straightforward, rapid, and accurate technique for assessing myocardial functions; thus, it is preferable for evaluating regional contractile function by determining the peak systolic strain [8].

A strong correlation has been recognized between the longitudinal strain and the left ventricular ejection fraction (LVEF) [8]. Additionally, the longitudinal strain quantitatively evaluates each LV segment of myocardial deformation, thereby promptly detecting systolic dysfunction in patients with preserved LVEF [9].

Real-time three-dimensional speckle-tracking echocardiography (3D STE) can be used to noninvasively and quantitatively assess global and regional myocardial wall motion. Current investigations demonstrated that strain and strain rate were preferable to wall motion analysis and LVEF in assessing myocardial systolic dysfunction [10].

A previous study demonstrated that 3D global peak longitudinal strain (GPLS) obtained from 3D STE technology can detect slight shifts in the longitudinal systolic function of the LV [11]. Thus, strain or strain rate may be useful in detecting complex coronary artery disease (CAD) in its early stages.

Distinguishing NSTE-ACS cases with a high chance of developing complex lesions early is critical to provide timely and optimal treatment. Nevertheless, global and regional longitudinal strain performance in evaluating the complex NSTE-ACS has not been thoroughly studied [10].

## Materials and methods

Subjects

The following procedures were performed on all patients.

Detailed Medical History

Within the first hour, the clinical data of patients were collected, including their cardiovascular risk factors, such as smoking, hypertension, diabetes mellitus (DM), age, gender, and symptom onset.

Full Physical Examination

This includes a general examination, in which all participants were subjected to a complete physical examination, including first medical contact vital signs, systolic blood pressure (BP) at presentation, and heart rate (HR), and local cardiac examination, including heart sounds, additional sounds, and cardiac murmurs.

Blood Samples

Venous blood samples were collected on admission before coronary angiography, and creatinine levels, lipid profile, and hemoglobin A1c (HbA1c) were measured.

Standard 12-Lead Electrocardiogram (ECG)

ECG is a necessary component of the diagnostic workup of all cases that is done within 10 minutes of initial contact with a medical professional to rule out ST-segment elevation myocardial infarction (STEMI).

Transthoracic Echocardiography (TTE)

TTE was done to detect longitudinal strains on a global and regional scale using 2D speckle-tracking imaging and then 3D. Echocardiographic data were acquired with an ultrasound Vivid E9 system (GE Vingmed Ultrasound AS, Horten, Norway), which was equipped with a two-dimensional 3.5-MHz transducer (M5S-D), three-dimensional 3.5-MHz transducer (4C-D), offline speckle-tracking analysis software, and background processing workstation (EchPAC BT 11.1.0, GE Medical System, Horten, Norway).

Estimation of longitudinal strain by 2D speckle-tracking: All participants were linked to the ECG and remained in the left lateral decubitus position throughout the study for the examination. Gated images were acquired with stable electrocardiographic traces during end-expiratory breath-holding, avoiding ventricle shortening, and allowing proper visualization of the endocardial border. The optimized frame rate is between 60 and 110 frames per second. Sector depth and width were kept to a minimum to concentrate the attention on the interest structure, three consecutive cardiac cycles were collected, and the final processing values were averaged. Apical three-chamber, four-chamber, and two-chamber views were obtained to detect/mark the reference landmarks (annulus and apex), trace the endocardial border, and adjust the region of interest width.

Offline analysis of the recordings was conducted using semiautomated computer software for strain and strain rate evaluation. Finally, this section provides regional and global longitudinal strain (GLS) values, which represent longitudinal strain averaged across all segments and views.

Measurement of longitudinal strain by 3D speckle-tracking: In the acquisition mode (3D full-volume mode), acquiring the entire apical volume in a single shot with clear endocardium was obtained by a 3D volumetric transducer with a frame rate between 18 and 25 frames per second. This larger pyramidal volume and subvolumes corresponding to 4-6 cardiac cycles in real time will be collected and stored. The dedicated software will calculate the 3D LV end-diastolic volume (LVEDV), LV end-systolic volume (LVESV), and regional and GLS.

Coronary angiography: Coronary angiography by radial approach was performed on all patients within 24 hours of admission (after echo image acquisition). Clinically significant stenosis was defined as a reduction in the left main coronary artery diameter by >50% or a diminution in the diameter of at least one major epicardial coronary artery or its main branches by >75%. The SYNTAX score was calculated after the angiographic procedure by two cardiologists, with experience performing interventional procedures, who are not aware of the study protocol or patient demographics. If scores differ, the final result was calculated using the mean values. Patients with a score of 22 or greater were classified as having severe and complex ACS. After getting the results of 2D and 3D strain echo and coronary angiogram of all study populations, the results of 2D and 3D regional and GLS to predict the severity and complexity of coronary lesions in terms of the SYNTAX score were compared, and the results of 2D and 3D longitudinal strain of different myocardial segments (regional longitudinal strain) and segmental coronary lesions by coronary angiography were correlated.

Ethical concerns: All patients provided written informed consent before participating in the study. Each patient was assigned a unique file with a unique code number. All investigations and findings were solely used for scientific purposes, with strict adherence to patient confidentiality. Unexpected risks that arose during the research were promptly communicated to the participants and the ethical committee.

Statistical method

The Statistical Package for the Social Sciences version 21 (IBM Corporation, Armonk, NY, USA) for Windows was utilized to analyze the data. Data were initially checked for normality using the Kolmogorov-Smirnov one-sample test.

Numbers and percentages were applied to describe the qualitative data. The Chi-square test was utilized to determine the association between categorical variables. Mean ± standard deviation (SD) was used for normally distributed data, while median (minimum-maximum) was used for non-normal data. The Student’s t-test was used for normal data and to compare the two groups, whereas the Mann-Whitney test was used for non-normal data.

Continuous data were correlated using the Spearman correlation. The receiver operating characteristic (ROC) curve was used to determine the sensitivity and specificity at various cutoff points.

Significance

The significance threshold was set at the 5% level (p-value) for all the abovementioned statistical tests, and the findings were considered significant when p < 0.05.

The lower the p-value, the more significant the findings will be. Significant variables are entered into a logistic regression model using statistical techniques to predict the most important determinants and account for potential interactions and confounding effects. The ROC curve was used to determine the sensitivity and specificity at various cutoff points.

## Results

Demographic data analysis revealed that the mean age distribution for all populations was 60.28 ± 10.92 years, and 76% were males and 24% were females.

**Figure 1:**
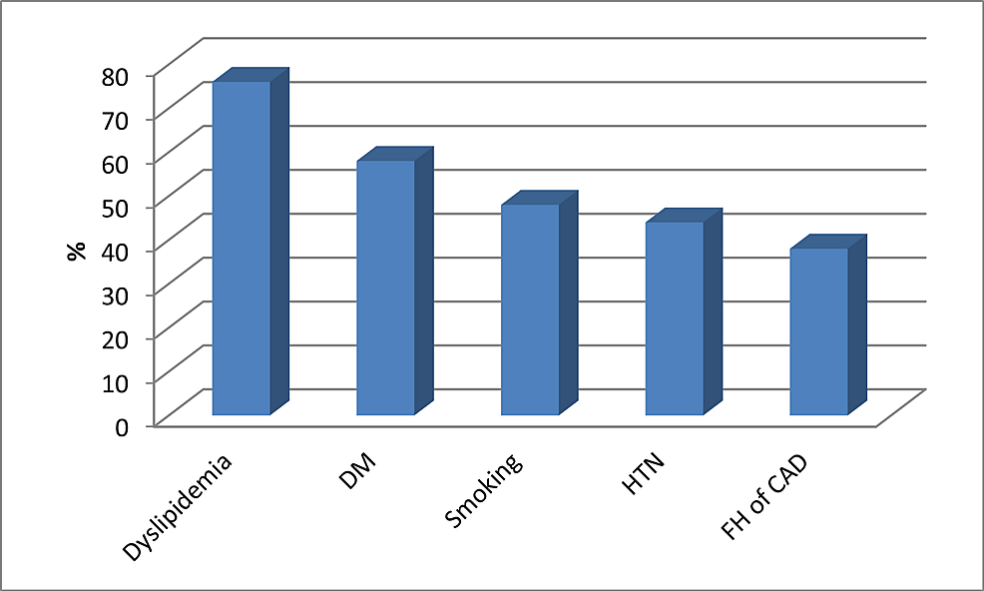
Risk factors among the study group DM: diabetes mellitus, HTN: hypertension, FM: family history, CAD: coronary artery disease

Risk factor evaluation revealed that 44% were hypertensive, 48% were smokers, 58% were diabetics, 76% had dyslipidemia, 50% had a BMI of >25 and 50% had a BMI of <25, and 38% had a family history of coronary heart disease as shown Table 1 and Figure 1.

**Table 1:** Demographic data and risk factors of the study group SD: standard deviation, DM: diabetes mellitus, HTN: hypertension, FH: family history, CAD: coronary artery disease

Variables	Study group (n = 100)
Age (years), mean ± SD	60.28 ± 10.92
Gender, male/female	76 (76%)/24 (24%)
DM	58 (58%)
HTN	44 (44%)
Smoking	48 (48%)
FH of CAD	38 (38%)
BMI, <25/>25	50 (50%)/50 (50%)
Dyslipidemia	76 (76%)

Table 2 shows the ECG changes among the study group, wherein 32% had nonsignificant changes, 32% had inverted T-wave, 10% had ST-segment depression, 9% had Q-waves and poor R-wave progression, 6% had bundle branch block (BBB), 5% had LV hypertrophy, 4% had biphasic T-wave, and 2% had sinus bradycardia and inverted T-wave.

**Figure 2:**
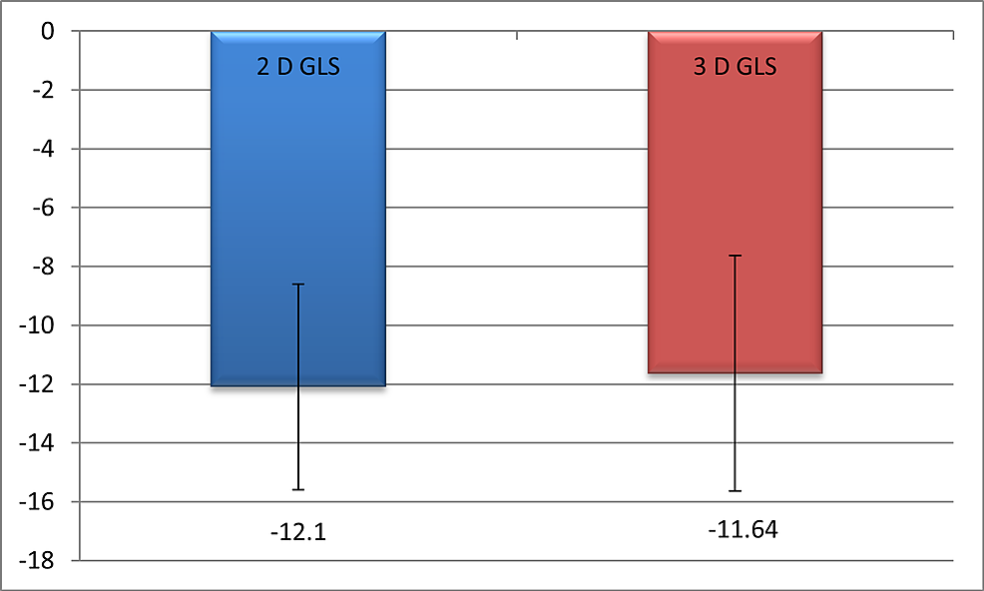
Comparison between 2D and 3D GLS GLS: global longitudinal strain

Table 3 shows the comparison between 2D and 3D GLS, with a mean of −12.10 ± 3.51 and −11.64 ± 4.05, respectively, with significant correlations (p ≤ 0.001) (Figure 2).

**Table 2:** ECG changes among the study group BBB: bundle branch block, LVH: left ventricular hypertrophy

ECG changes	Study group (n = 100)
No significant changes	32 (32%)
Inverted T-wave	32 (32%)
ST-segment depression	10 (10%)
Q-waves and poor R-wave progression	9 (9%)
BBB	6 (6%)
LVH	5 (5%)
Biphasic T-wave	4 (4%)
Sinus bradycardia and inverted T-wave	2 (2%)

**Table 3:** Comparison between 2D and 3D GLS SD: standard deviation, GLS: global longitudinal strain *A p-value of 0.05 or less is considered statistically significant.

	2D GLS	3D GLS	Paired t-test	p-value
Mean ± SD	−12.10 ± 3.51	−11.64 ± 4.05	4.25	≤0.001*

Table 4 shows the 3D echo parameters, with a mean of −11.04 ± 2.78 for GCS, 22.32 ± 9.99 for GRS, −17.97 ± 5.43 for area strain, and 47.58 ± 11.98 for 3D EF.

**Table 4:** 3D echo parameters among the study population GCS: global circumferential strain, GRS: global radial strain, EF: ejection fraction

	Study group (n = 100)
GCS	−11.04 ± 2.78
GRS	22.32 ± 9.99
Area strain	−17.97 ± 5.43
3D EF	47.58 ± 11.98

Table 5 shows the significant correlation between 2D and 3D GLS with p-values of 0.017 and ≤0.001, respectively. The right coronary artery (RCA) had a nonsignificant correlation between 2D and 3D GLS with a p-value of 0.429.

**Table 5:** Comparison between 2D and 3D regional longitudinal strain LAD: left anterior descending coronary artery, LCX: left circumflex artery, RCA: right coronary artery *A p-value of 0.05 or less is considered statistically significant.

	2D regional longitudinal strain	3D regional longitudinal strain	Paired t-test	p-value
LAD territory	−11.13 ± 4.47	−10.84 ± 5.18	2.44	0.017*
LCX territory	−12.54 ± 4.11	−12.05 ± 4.29	4.72	≤0.001*
RCA territory	−12.67 ± 4.06	−12.55 ± 4.63	0.793	0.429

The LM was affected in 16% of the study population, the LAD in 58%, the left circumflex artery (LCX) in 53%, and the right coronary artery (RCA) in 46%. Normal or nonsignificant lesion was observed in 24%, single-vessel disease in 26%, double-vessel disease in 12%, and triple-vessel disease in 38%, as shown in Table 6.

**Table 6:** Vessels affected among the study group LM: left main, LAD: left anterior descending coronary artery, LCX: left circumflex artery, RCA: right coronary artery

Vessels affected	Study group (n = 100)
LM	16 (16%)
LAD	58 (58%)
LCX	53 (53%)
RCA	46 (46%)
Number of vessels affected, normal or nonsignificant/single vessel/two vessels/triple vessels	24 (24%)/26 (26%)/12 (12%)/38 (38%)

Regarding speckle-tracking echo parameters between both groups, 3D EF, 2D GLS, 3D GLS, GCS, GRS, and area strain all had a significant correlation between low and high SYNTAX score groups with a p-value of ≤0.001 for all, as shown in Table 7.

**Table 7:** Speckle-tracking echo parameters between both groups EF: ejection fraction, GLS: global longitudinal strain, DGLS: delta global longitudinal strain, GCS: global circumferential strain, GRS: global radial strain *A p-value of 0.05 or less is considered statistically significant.

	Low score (n = 36)	Intermediate and high score (n = 37)	Test of significance	p-value
3D EF	53.72 ± 7.43	36.16 ± 9.40	t = 8.83	≤0.001*
2D GLS	−14.03 ± 1.83	−8.28 ± 1.96	t = 12.91	≤0.001*
3 DGLS	−14.04 ± 2.23	−7.16 ± 2.02	t = 13.78	≤0.001*
GCS	−12.50 ± 1.82	−8.65 ± 2.27	t = 7.97	≤0.001*
GRS	24.22 ± 13.17	16.32 ± 5.26	t = 3.38	≤0.001*
Area strain	−20.81 ± 3.20	−12.19 ± 2.98	t = 11.88	≤0.001*

In the comparison of both groups in terms of the affected vessel, 5.6%, 55.6%, 55.6%, and 33.3% of the LM, left anterior descending coronary artery (LAD), LCX, and RCA, respectively, were affected in group I, whereas 37.8%, 100%, 86.5%, and 86.5, respectively, were affected in group II, with significant p-values of 0.001, ≤0.001, 0.004, and ≤0.001, respectively. Regarding the number of affected vessels, a significant correlation was found between both groups (p ≤ 0.001) (Table 8).

**Table 8:** Relation between the SYNTAX score and affected vessels LM: left main, LAD: left anterior descending coronary artery, LCX: left circumflex artery, RCA: right coronary artery *A p-value of 0.05 or less is considered statistically significant.

	Low score (n = 36)	Intermediate and high score (n = 37)	Test of significance	p-value
LM	2 (5.6%)	14 (37.8%)	c^2^ = 11.11	0.001*
LAD	20 (55.6%)	37 (100%)	c^2^ = 21.06	≤0.001*
LCX	20 (55.6%)	32 (86.5%)	c^2^ = 8.52	0.004*
RCA	12 (33.3%)	32 (86.5%)	c^2^ = 21.53	≤0.001*
Number of vessels, single vessel/two vessels/triple vessels	24 (66.7%)/6 (16.7%)/6 (16.7%)	0 (0%)/6 (16.2%)/31 (83.8%)	c^2^ = 40.88	≤0.001*

Table 9 shows the statistically significant correlation between the SYNTAX score and some variables.

**Table 9:** Correlation between the SYNTAX score and other variables s.Cr: serum creatinine, LDL: low-density lipoprotein, HDL: high-density lipoprotein, EF: ejection fraction, GLS: global longitudinal strain, GCR: global circumferential strain, GRS: global radial strain, LAD: left anterior descending coronary artery, LCX: left circumflex artery, RCA: right coronary artery *A p-value of 0.05 or less is considered statistically significant.

Variables	SYNTAX score	
	r	p-value
Age	−0.103	0.384
HbA1c	0.562	≤0.001*
s.Cr	0.310	0.008*
Troponin	0.224	0.055
LDL	0.298	0.010*
HDL	−0.264	0.023*
EF	−0.793	≤0.001*
2D GLS	0.908	≤0.001*
3D GLS	0.946	≤0.001*
GCS	0.809	≤0.001*
GRS	−0.375	0.001*
Area strain	0.872	≤0.001*
LAD territory	0.863	≤0.001*
LCX territory	0.581	≤0.001*
RCA territory	0.765	≤0.001*
LAD territory	0.875	≤0.001*
LCX territory	0.627	≤0.001*
RCA territory	0.837	≤0.001*
Number of vessels	0.814	≤0.001*

**Figure 3:**
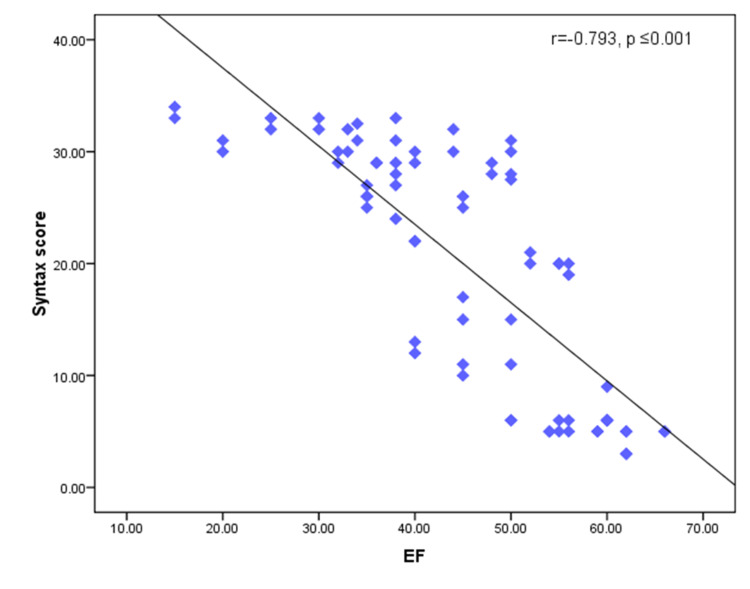
Scatter diagram for the negative correlation between the SYNTAX score and EF EF: ejection fraction

**Figure 4:**
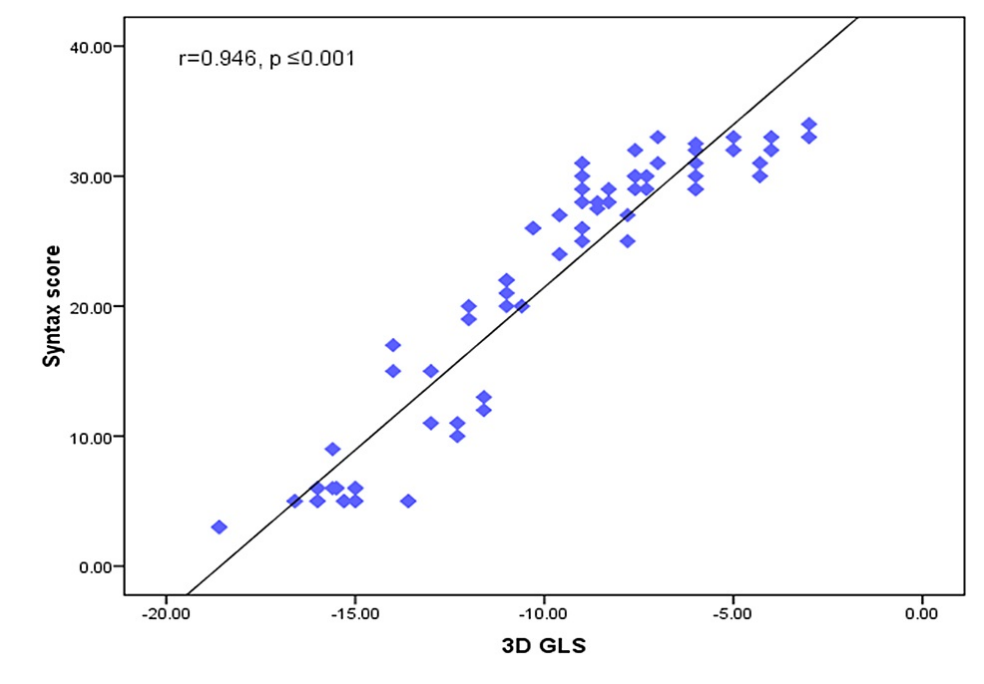
Scatter diagram for the positive correlation between the SYNTAX score and 3D GLS GLS: global longitudinal strain

**Figure 5:**
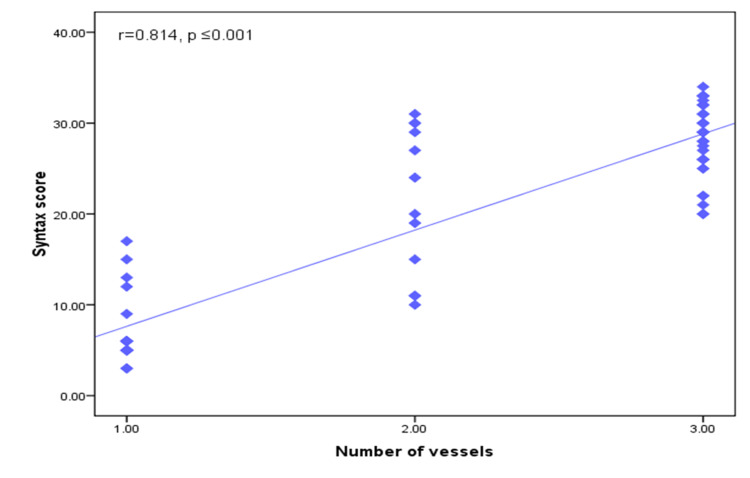
Scatter diagram for the positive correlation between the SYNTAX score and the number of affected vessels

There was a positive significant correlation between the SYNTAX score HbA1c, s.Cr, LDL, 2D GLS, 3D GLS, global circumferential strain (GCS), area strain, 2D LAD, 2D LCX, 2D RCA, 3D LAD, 3D LCX, and 3D RCA territories and the number of affected vessels (p-values of ≤0.001, 0.008, 0.010, ≤0.001, ≤0.001, ≤0.001, 0.001, ≤0.001, ≤0.001, ≤0.001, ≤0.001, ≤0.001, ≤0.001, ≤0.001, and ≤0.001, respectively (Figures 3-5).

A negative significant correlation was found in HDL, EF, and GRS (p-values of 0.023, ≤0.001, and 0.001, respectively).

**Figure 6:**
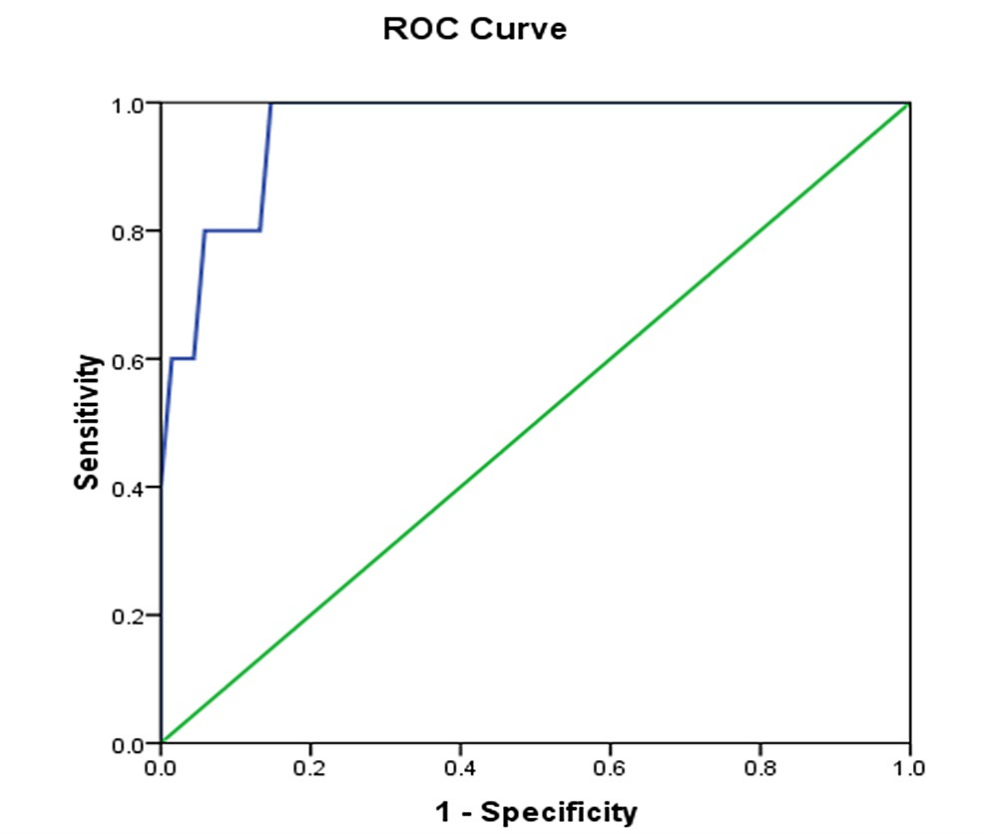
ROC curve to predict intermediate and high SYNTAX scores using 3D GLS GLS: global longitudinal strain

The best cutoff value considering 3D GLS in intermediate prediction and high SYNTAX score is −5.5 with 80% sensitivity, 94.1% specificity, 50 positive predictive value, 98.5 negative predictive value, and 0.96 area under the curve (AUC), as shown in Table 10 and Figure 6.

**Table 10:** Receiver operating characteristic (ROC) curve to predict intermediate and high SYNTAX scores using 3D GLS GLS: global longitudinal strain, AUC: area under the curve, CI: confidence interval, PPV: positive predictive value, NPV: negative predictive value

AUC	95% CI		Cutoff	Sensitivity	Specificity	PPV	NPV	Accuracy
	Lower	Upper						
0.96	0.90	1.0	−5.50	80%	94.1%	50	98.5	93.2%

## Discussion

A rapid evaluation of patients with severe and complex CAD, such as a left main lesion and/or three-vessel disease, is essential to determine the prognosis and select an appropriate NSTE-ACS treatment approach. The recently issued clinical guidelines for NSTE-ACS management strongly recommend initiating dual antiplatelet therapy as soon as possible, with aspirin and P2Y12 inhibitors, including clopidogrel, ticagrelor, or prasugrel [12].

However, in patients undergoing preoperative CABG surgery, a combination of antiplatelet therapy may raise the possibility of perioperative bleeding complications. Therefore, clinicians may choose to discontinue clopidogrel in cases in which CABG surgery is likely to be required. The prompt initiation of dual antiplatelet therapy would necessitate postponing CABG surgery due to intraoperative bleeding concerns. In contrast, delaying the P2Y12 inhibitor treatment may increase fatal events in patients with NSTE-ACS [3].

Thus, an early (i.e., before angiography) indicator capable of cost-effectively evaluating complex and severe coronary artery symptoms is required to efficiently instruct our subsequent treatment [12].

The SYNTAX trial established the synergy between cardiac surgery (SYNTAX score) and percutaneous coronary intervention with Taxus. In 2005, it was described as “a coronary angiographic tool to measure CAD based on its complexity” [4].

The SYNTAX score is a diligent angiographic scoring system based on the complexity of lesions in the coronary arteries. The SYNTAX score can be used to determine the degree of coronary artery stenosis and evaluate the presence of coronary artery calcification and tortuosity-, bifurcation-, or trifurcation-type lesions. Nonetheless, it is a minimally invasive procedure that utilizes coronary angiography [5,13].

Real-time 3D STE can be used to noninvasively and quantitatively determine the global and regional motion of the myocardial wall. This technology has been compared to that of MRI tagging [14].

Earlier research demonstrated that 3D GPLS originates from 3D STE technology and is capable of detecting subtle changes in the longitudinal systolic function of the LV. Thus, strain or strain rate may be beneficial in the prompt detection of complicated CAD. It is critical to promptly distinguish NSTE-ACS cases with a high probability of developing complex lesions to provide treatment that is both timely and optimal. However, the effectiveness of GPLS in evaluating the difficult NSTE-ACS has not been thoroughly investigated [11].

Therefore, this research was conducted to compare 2D and 3D regional longitudinal strain in assessing coronary artery lesions that are intricate in 100 patients with NSTE-ACS using the SYNTAX score as a reference standard.

All patients were evaluated for a complete history and physical examination, HbA1c, lipid profile, and creatinine assessment, 12-lead ECG, and TTE to measure the global and regional longitudinal strain using 2D speckle-tracking imaging, followed by 3D and SYNTAX score calculation.

The participants in the current study have a mean age of 60.28 ± 10.92 years, with the majority being male (76%). Of the cases, 58% had DM, 44% had HTN, 76% had dyslipidemia, and 48% was a smoker. Additionally, 38% had a positive family history of CAD, and a BMI of either <50 or >50 was equally distributed.

In agreement with the current study, Lapu-Bula et al. evaluated the potentially related factors to NSTEMI and reported a modestly significant positive correlation between NSTEMI and elevated systolic blood pressure (BP). Other clinical factors, such as a history of HTN or CAD, smoking, and DM, were not associated with NSTEMI [15].

Similarly, Ralapanawa et al. reported that HTN was significantly more prevalent in patients with NSTEMI. Their study also showed that smoking, a family history of ACS, DM, HTN, all types of ACS, and dyslipidemia were prevalent [16].

Additionally, Brunori et al., in their cross-sectional study on 150 patients admitted to the hospital due to acute coronary syndrome, revealed that the overwhelming majority of patients (44.6%) were overweight, followed by those with normal BMI (33.3%), those who were obese (21.4%), and those who were underweight (0.7%) [17].

In harmony with our work, Radwan et al., in their study, included 96 patients with NSTEMI and demonstrated a male predominance (66.7 %) in the study population, as well as an elevated prevalence of dyslipidemia (69%), DM (65%), smoking (56%), and HTN (52%) [18].

In the current study, the mean value of HbA1c was 7.86 ± 2.32, s.Cr was 0.988 ± 0.35, troponin was 2.3 K0 (normal range: 0.0-45), LDL was 179.24 ± 66.01, and HDL was 22.94 ± 10.94.

Brunori et al. revealed that the mean LDL values were either close to or exceeded the established norms; however, the mean HDL level was low [17]. Moreover, Galal et al. demonstrated that the NSTE-ACS group had significantly higher levels of total LDL-C [19].

In the current study, 32 (32%) cases had no significant changes or inverted T-wave, 10 (10%) had ST-segment depression, nine (9%) had Q-waves and poor R-wave progression, six (6%) had BBB, five (5%) had LVH, four (4%) had biphasic T-wave, and two (2%) had sinus bradycardia and inverted T-wave.

Similarly, Birnbaum et al. concluded that alterations in the ECG are found in many patients; these changes are often minimal or reflect a reperfusion state (inversion of the terminal, T-wave portion, or nonspecific T-wave changes without significant ST-segment deviation). Numerous distinct ECG patterns have been identified in cases with NSTE-ACS as T-wave inversion with isoelectric ST segments or with a minor ST deviation. Generally, isolated T-wave inversion is not regarded as a concerning sign in NSTE-ACS. Flat/mild isolated T-wave inversion, particularly in leads with prominent R-waves, is frequently observed following the resolution of symptoms and is regarded as a sign of reperfusion [20].

Strain variations assist in determining ischemic myocardium during rest and stress echocardiography and may provide prognostic information. Additionally, it may aid in defining the magnitude of myocardial infarction transmural and the presence of viable myocardium. Ischemic myocardium exhibits decreased or absent regional systolic longitudinal and circumferential shortening, as well as radial thickening [21]. In the current study, the GCS was −11.04 ± 2.78, GRS was 22.32 ± 9.99, area strain was −17.97 ± 5.43, and 3D EF was 47.58 ± 11.98. This was the first study that compared 2D and 3D in NSTEMI.

This difference was explained by the validation of 2D STE for assessing myocardial deformation, while 3D STE has been recently lauded as a potentially more fruitful technique for precisely assessing segmental and global LV function. Since the foreshortened views did not affect 3D STE, avoiding the weakness of out-of-plane motion that occurs as the heart enters and exits the incident imaging plane reduces or eliminates the ability to track the same speckle throughout the heart cycle. Additionally, 3D STE required only a single apical four-chamber view to conduct all assessments [22].

In line with our work, Radwan et al. reported that GLS values ranged from −14.21 to −17.87 with a mean of −15.8 ± 0.83 in 96 patients with NSTEMI. The GCS value ranged from −25.65 to −33.93 with a mean of −31.2 ± 1.9 [18].

Additionally, Caspar et al. revealed that GLS was significantly more numerically altered in cases with three-vessel disease (−15% ± 2.3%) than in those with one-vessel disease (−17.3% ± 3.7%) or two-vessel disease (−16.6% ± 2.8%); however, none of these differences were statistically significant [23].

In the current study, a significantly higher value was found in the GLS, LAD territory, and LCX territory using 2D regional longitudinal strain (−12.10 ± 3.51, −11.13 ± 4.47, and −12.54 ± 4.11, respectively) than that using the 3D regional longitudinal strain (−11.64 ± 4.05, −10.84 ± 5.18, and −12.05 ± 4.29, respectively) (p-values of 0.001, 0.017, and <0.001, respectively). Additionally, no significant changes were found in the RCA territory (p = 0.429).

Similarly, Abd Allah et al. revealed that the territorial strain of the LAD was significantly (p = 0.05) greater than those with nonsignificant LAD lesions (−17.6% + 3.5%) and those with significant LAD lesions than the control group (−20% + 2.1%). In severe RCA disease, the territorial strain values were −13.7% + 4.2%, which was significantly higher (p = 0.05) than those with nonsignificant RCA lesions (−17.6% + 3.9%) and in comparison to the control group (−19.6% + 3%). Lastly, the territorial strain of the LCX was accompanied by significant (p < 0.05) reductions in mortality compared to those with nonsignificant LCX lesions (−16.7% + 4.8%) or the control group (−20.2% + 2.3%) [24].

Similarly, Choi et al. demonstrated that resting GLS was significantly lower in cases with severe CAD (three-vessel/left main CAD), which did not have resting echocardiographic regional wall motion abnormalities than in cases with CAD that involve one or two vessels [25].

In the current study, the LAD was affected in the majority of cases (58 (58%)), followed by the LCX in 53 (53%), RCA in 46 (46%), and LM in 16 (16%). According to the number of affected vessels, 38 (38%) had a triple-vessel disease, 26 (26%) had a double-vessel disease, 24 (24%) had normal/nonsignificant lesion, and 12 (12%) had a single-vessel disease.

This result was supported by Brunori et al., who revealed a greater proportion of patients (69.3 %) who had one or two affected coronary arteries [17].

However, the study by Sidhu et al. revealed that 81% of patients underwent coronary angiography. The most prevalent pattern of coronary artery association (43.3%) was a single-vessel disease, with the LAD as the most frequently involved vessel (62.8%). The RCA was the second most frequently involved vessel, with 202 (40.2%) patients. In our study, the LCX has affected 161 (32%) cases [26].

According to Vilela, 100 patients with NSTE-ACS underwent coronary angiography, of which 48% had a 70% lesion in the LAD, 36% had a 70% lesion in the circumflex coronary artery, and 27% had a 70% lesion in the RCA. Eleven of those cases exhibited collateral circulation, whereas the remaining had an occluded distal bed [27].

Conversely, Tanaka et al. revealed that one-vessel disease was the most prevalent type, affecting 148 (41.2 %) subjects. Three-vessel disease was the least prevalent (19.2%). However, their results were similar to our findings in terms of the target vessels for percutaneous coronary intervention; the LAD was the most frequently encountered, accounting for 281 (52.3%) lesions [28].

In the current study, the median SYNTAX score among the study group was 24.5 (33.4%), while 36 (49.3%) had a lower score, of which 32 (43.8%) had an intermediate score, and five (6.8%) had a high score.

Our results were higher than that of Tanaka et al., with a mean SYNTAX score of 19.1 ± 11.4 [28].

Additionally, Cai et al. enrolled 59 inpatients with NSTE-ACS and revealed a mean SYNTAX score of 19.52 ± 8.09, and 27 (45.8%) patients had three-vessel disease (including left main lesion). GPLS had a mean value of −12.24% ± 3.50% [12].

Additionally, according to the study of Obeid et al., the total number of patients stratified by SxSIILow, SxSIIMid, and SxSIIHigh tertiles was 245, 245, and 244, respectively. The mean SYNTAX score for anatomical characteristics was 17.56 ± 9.3, with a median of 16 and an interquartile range (IQR) of 13 [29].

In the current study, there was a significantly lower 3D EF, 2D GLS, 3D GLS, GCS, GRS, and area strain and lower 2D LAD, 2D LCX, 2D RCA, 3D LAD, 3D RCA, and 3D LCX territories in the intermediate and high score group than in the low score group of patients (p < 0.001 for all).

Similarly, Cai et al. revealed that LVESV, LVEF, and GPLS were significantly associated with the SYNTAX score (p < 0.001) [12].

In line with the current work, Cho et al. reported that patients with low SYNTAX score had a significantly greater EF compared to those with a higher score (60.4% ± 10.6% with low, 58.1% ± 11.3% with intermediate, and 56.6% ± 11.3% with a high score; p = 0.025) [30].

Moreover, Serdar Kuyumcu et al. revealed that the high SYNTAX score group (score of ≥32) had significantly higher rates of collateral vessels [31].

Parallel findings have been reported by Radwan et al., wherein the GLS of the apical inferior, mid-anterior, basal inferior, and mid-inferior were significantly decreased in the high-risk group (HRG), while the group with low-intermediate risk demonstrated a significant reduction in the LS of the mid-anterolateral, apical lateral, and apex. Generally, the GLS demonstrated a statistically significant (p = 0.02) reduction in the high-risk group (15.4 ± 0.6) compared to the low-intermediate-risk group (16 ± 0.8) [18].

Additionally, Fahim et al. classified 20 (24.7%) patients into the normal coronary group, 27 patients (33.3%) with one-/two-vessel CAD corresponding to the low-risk group (LRG), and 34 (42%) patients with three-vessel/left main CAD reflecting the HRG. In the HRG, GLS, global longitudinal strain rate (GLSR), global radial strain rate (GRSR), mid-circumferential strain (MCS), and mid-circumferential strain rate (MCSR) were significantly lower than in the LRG (p = 0.030, p = 0.009, p = 0.000, p = 0.000, and p = 0.004, respectively) [32].

The current study revealed a significantly higher incidence of affected LM, LAD, LCX, and RCA in patients with intermediate and high scores than those with low scores (p < 0.05). Additionally, the number of affected vessels was significantly higher in patients with intermediate and high scores than in those with low scores (p < 0.001).

Cho et al. reported significantly fewer multiple-vessel lesions in cases with lower SYNTAX scores (75.1% with low, 93% with intermediate, and 98.8% with a high score; p < 0.0001) [30].

Additionally, Obeid et al. reported that the angiographic characteristics revealed significant differences, including a higher rate of microvascular decompression in patients with LAD involvement (74.2%) in the SxSII high tertile compared with cases in the lower tertiles [29].

Moreover, Serdar Kuyumcu et al. revealed that the high SYNTAX score group (score of ≥32) had significantly higher rates of coronary artery involvement with multiple vessels and chronic total occlusion compared with the low SYNTAX score group (score of <32) [31].

The current study revealed a significant positive relationship between the SYNTAX score and HbA1c, s.Cr, and LDL; 2D GLS, 3D GLS, and GCS; LAD, LCX, and RCA territories; and the number of vessels. A significant negative relationship was found between the SYNTAX score and HDL, EF, and GRS; however, no significant relationship was found between the SYNTAX score and age and troponin.

Similarly, when Vrettos et al. examined 71 cases, they discovered that GLS values were inversely related to SYNTAX score values. In the low SYNTAX score group, this correlation was weaker (r2 = 0.1332, p < 0.05), whereas it was lost in the high SYNTAX score group (r2 = 0.0002, p = NS) [33].

The current study revealed a cutoff of −5.50 and AUC of 0.96, and 3D GLS had 80% sensitivity and 94.1% specificity with 93.2% accuracy for predicting intermediate and high SYNTAX scores. This demonstrated that the GPLS may be superior to conventional echocardiography and stress testing in diagnosing coronary disease.

Similarly, Vrettos et al. demonstrated that the optimal GLS cutoff value for identifying patients with a high SYNTAX score was −13.95 (sensitivity of 71% and specificity of 90%; p < 0.001) [33].

Moreover, Cai et al. revealed that by comparing the GPLS score to the SYNTAX score, the analysis of the ROC curve demonstrated the value of GPLS in diagnosing severe and complex NSTE-ACS. The AUC was 0.882 (p < 0.001). The ROC curve analysis demonstrated that the optimal cutoff value for GPLS in the diagnosis of severe and complex NSTE-ACS was −11.76%, with a sensitivity of 82.6% and a specificity of 83.3% [12].

Additionally, Choi et al. revealed that 2D STE-derived resting GPLS could be used to monitor patients with left main and/or three-vessel CAD. They demonstrated that a cutoff value of −11.76% was the optimal value for evaluating patients with a SYNTAX score of intermediate or high, with a sensitivity of 82.6% and a specificity of 83.3% [25].

Moreover, Caspar et al. revealed a high GLS diagnostic performance for the prediction of CAD in the ROC curve analysis (AUC = 0.92), with a sensitivity of 81% and a specificity of 88% at the optimal cutoff of −19.7%. When the GLS cutoff value was raised to −21%, the sensitivity increased to 100% with a specificity of 68% [23].

The study by Abdelrazek et al., using a GLS cutoff value of 17.8%, expected a low SYNTAX score with 84% sensitivity and 70% specificity. In contrast, a GLS cutoff value of 15.9% postulated a rising SYNTAX score, with a sensitivity of 97.1% and a specificity of 90% [34].

## Conclusions

This study was conducted in response to the demand for a noninvasive and simple method of identifying patients with CAD. We hypothesized that global longitudinal peak systolic strain (GLPSS) is associated with CAD severity. This study aimed to investigate this hypothesis-generating concept, which has the potential to improve patient selection for coronary angiography. Our findings indicate that GLPSS can assist in identifying patients with severe CAD manifested by a high SYNTAX score.

The current study establishes that 2D and 3D STE are noninvasive, reproducible, and effective techniques, which may be clinically used to evaluate coronary lesions in patients with NSTE-ACS. They can be routinely used to diagnose and stratify patients at high risk of NSTE-ACS, thereby resulting in improved patient management.
